# Effects of the COVID-19 Pandemic on the Mental Health of Healthcare Providers: A Comparison of a Psychiatric Hospital and a General Hospital

**DOI:** 10.3389/fpsyt.2021.720693

**Published:** 2022-01-14

**Authors:** Daniel Anzola, Jacqueline Limoges, Jesse McLean, Nathan J. Kolla

**Affiliations:** ^1^Department of Research and Innovation, Georgian College, Barrie, ON, Canada; ^2^Department of Health Wellness and Sciences, Georgian College, Barrie, ON, Canada; ^3^Faculty of Health Disciplines, Athabasca University, Athabasca, AB, Canada; ^4^Centre for Education and Research, Royal Victoria Regional Health Centre, Barrie, ON, Canada; ^5^Waypoint Centre for Mental Health Care, Penetanguishene, ON, Canada; ^6^Waypoint/University of Toronto Research Chair in Forensic Mental Health Science, Penetanguishene, ON, Canada; ^7^Centre for Addiction and Mental Health, Toronto, ON, Canada; ^8^Department of Psychiatry, University of Toronto, Toronto, ON, Canada

**Keywords:** COVID-19, pandemic, healthcare provider, psychological distress, anxiety

## Abstract

**Background:**

Before the COVID-19 pandemic, healthcare providers (HCPs) were already experiencing a higher prevalence of mental health disorders compared with non-healthcare professionals. Here, we report on the psychosocial functioning and stress resilience of HCPs who worked during the COVID-19 pandemic in a large-sized psychiatric facility and a large acute care hospital, both located in central Ontario, Canada.

**Methods:**

Participants completed five validated psychometric instruments assessing depression, anxiety, and stress (The Depression, Anxiety, and Stress Scale-21, DASS-21); work-related quality of life (Work-Related Quality of Life Scale, WRQoL); resilience (Connor-Davidson Resilience Scale, CD-RISC); anxiety about the novel coronavirus (Coronavirus Anxiety Scale, CAS); and loneliness (UCLA Loneliness Scale, ULS). Participants from the psychiatric hospital (*n* = 94) were sampled during the easing of restrictions after the first wave in Ontario, and participants from the acute care hospital (*n* = 146) were sampled during the height of the second wave in Ontario.

**Results:**

Data showed that HCPs from the acute care hospital and psychiatric hospital reported similar scores on the psychometric scales. There were also no significant differences in psychometric scale scores between medical disciplines at the acute care hospital. Among all HCPs, being a nurse predicted better quality of life (*p* = 0.01) and greater stress resilience (*p* = 0.031).

**Conclusion:**

These results suggest that HCPs' psychological symptoms are similar across the hospital settings sampled. Compared to other HCPs, nurses may show a unique resiliency to the pandemic. We suggest that emergencies such as the COVID-19 pandemic have a pervasive effect on HCPs. It is important to address HCPs' mental health needs in terms of crisis management and improve resilience among all HCPs during the inter-crisis period before a new challenge arrives.

## Introduction

The first cases of coronavirus disease 2019 (COVID-19), caused by the novel severe acute respiratory syndrome coronavirus 2 (SARS-CoV-2), were reported in Wuhan, Hubei Province, China, between December 2019 and January 2020 (1). COVID-19 quickly became a global pandemic (2), was declared a global public health emergency in February 2020 by the World Health Organization (3), and by April 2021, the death toll in Canada had reached 23,315 persons (4). To combat COVID-19 transmission, Canadian federal and provincial governments began implementing public health measures mid-March 2020, including restrictions on group gatherings, border closures and restricted travel, school/childcare closures, work from home mandates, and temporary suspension of non-essential health and public services (5). The pandemic and the infection control measures put in place to curb COVID-19 brought new mental health challenges to the general population in Canada, caused by physical distancing measures, social isolation, financial and employment insecurity, housing instability, and changes to health and social care access. These factors all contributed to a broadening of mental health inequities (6, 7). Surveys conducted by Statistics Canada and the Angus Reid Institute during the first semester of 2020 reported that Canadians perceived a deterioration of their mental health, as well as an increase in their consumption of alcohol, cannabis, and tobacco (6, 8, 9).

Before the COVID-19 pandemic, HCPs were already experiencing a higher prevalence of mental health disorders compared with non-healthcare professionals (10–12). A survey conducted in 2019 of United States physicians identified alarmingly high rates of self-reported burnout (44%), suicidal thoughts (14%), and suicide attempts (1%) (13). As would be expected, the extra social- and work-related stressors from COVID-19 have made the situation more critical for HCPs: by early 2020, reports already indicated that the increased complexity and challenges that HCPs faced while confronted with the contagion, including lack of availability of personal protective equipment in some jurisdictions (2, 14), were negatively affecting their mental health. HCPs started showing burnout (15, 16) and higher levels of depression, anxiety, and stress (17, 18), and reports of HCPs in China working with COVID-19 revealed a high prevalence of insomnia, anxiety, depression, somatization, and obsessive-compulsive symptoms (18, 19). Nearly one in five nurses and more than one in seven clinicians in intensive care units reported thoughts of self-harm or suicide (18).

Other studies have highlighted the possibility of workforce disruptions in nursing professionals when their mental health is overlooked (20) and have pointed to the similarities between the COVID-19 pandemic and previous viral outbreaks and disasters that increased psychiatric morbidity in this population (21). Moreover, a systematic review published in 2021 of European and American samples reported moderate and high levels of stress, anxiety, depression, sleep disturbance, and burnout among HCPs working with COVID-19 patients (22).

Given the challenging circumstances that HCPs are facing working in the COVID-19 pandemic that can adversely affect their psychosocial functioning, our study sampled HCPs at a large-sized mental health care facility in rural Ontario, Canada, and a large acute care hospital in Ontario, Canada, in an urban setting. Data were collected during the easing of restrictions in summer 2020 at the psychiatric hospital and during the height of the second wave in the general hospital. We had two specific objectives: first, to compare measures of resilence and psychosocial functioning obtained from the psychiatric HCPs versus scores obtained from HCPs working at the general hospital; and second, to discern whether differences in scores existed between different types of psychiatric and medical services offered at the general hospital (e.g., HCPs in internal medicine versus those in surgery). We additionally sought to identify predictors of quality of life and stress resilience among participants from both sites. This study provides information about two different points in time during the COVID-19 pandemic and two different health care delivery settings.

## Methods

This study was approved by the Research Ethics Boards of the Royal Victoria Regional Health Centre (RVH), Waypoint Centre for Mental Health Care (Waypoint), and Georgian College. All participants provided informed consent through an online form. A gift card for $20 CAD was offered as a token of appreciation to study participants.

### Setting

Study participants were recruited from Waypoint, a 301-bed psychiatric hospital located in Penetanguishene, Ontario, and RVH, a 408-bed acute care community hospital located in Barrie, Ontario.

### Participants

The study included HCPs employed at Waypoint and RVH. All actively employed HCPs were eligible to participate in the study, including physicians, nurses, and allied healthcare professionals. Importantly, data from Waypoint were collected during the easing of restrictions following the first wave of the pandemic in Ontario (August 18–27, 2020), while data from RVH were collected during the height of the second wave of the pandemic when strong public health measures were in effect (December 22, 2020–February 9, 2021).

### Instruments and Survey Design

An online data collection tool was designed to capture demographic information, general information about living conditions, and deliver five self-report instruments that would provide quantitative data relevant to mental health functioning during the pandemic. The data were analyzed using IBM SPSS V25.

To identify anxiety associated with the COVID-19 pandemic, we used the *Coronavirus Anxiety Scale* (CAS), a recently developed self-report, 5-item scale that assesses anxiety symptoms related to COVID-19. It is a short instrument, where participants rate on a 0 (not at all) to 4 (nearly every day over the last 2 weeks) scale how frequently they experienced coronavirus anxiety (e.g., “I felt paralyzed or frozen when I thought about or was exposed to information about the coronavirus.”). It has high internal consistency (Cronbach's alpha = 0.93) (23, 24).

Depression, anxiety, and stress symptoms were evaluated using the *Depression, Anxiety*, and *Stress Scale-21* (DASS-21), a widely used and validated 21-item scale with three domains to measure the degree of stress, depression, and anxiety (25). Its internal consistency is high (Chronbach's alphas = 0.91, 0.80, and 0.84 for the Depression, Anxiety, and Stress sub-scales, respectively) (26).

To assess work-related quality of life, we used the *Work-Related Quality of Life Scale* (WRQoL), one of the most succinct (23 self-report items) yet psychometrically valid and reliable scales assessing quality of work-life. Its use has been validated to assess HCPs (27), and it is a fully tested, comprehensive, psychometric measure of an employee's quality of working life. It has high internal consistency (Cronbach's alpha = 0.91) (28).

We evaluated loneliness using the *UCLA Loneliness Scale* (ULS), a 20-item instrument that measures how frequently a person feels disconnected from others. It has been validated in a variety of populations, including HCPs. It has high internal consistency (Cronbach's alpha = 0.89 to 0.94) (29).

Finally, to assess resilience, we used the *Connor-Davidson Resilience Scale* (CD-RISC), a widely used and validated measure of stress resilience that demonstrates superior psychometric properties. Scores range from 0 to 100, where lower scores are indicative of greater stress intolerance (30). It has high internal consistency (Cronbach's alpha = 0.92) (31).

### Data and Statistics

In total, 240 participants (94 from Waypoint and 146 from RVH) completed the study. Demographic characteristics between the sites were compared using chi-square tests. Pearson's correlations were computed to compare the associations between scale scores. For the general hospital data, analysis of variance (ANOVA) was used to assess if there were significant differences in scale scores between the different medical departments at RVH for each scale. Finally, stepwise multiple linear regression was employed to identify significant predictors of quality of life and stress resilience among all HCPs.

Approximately 800 HCPs are employed at Waypoint and 2,500 at RVH. Email invitations were sent to all HCPs through the employee email distribution lists. Links to the data collection tool were posted in internal communications (e.g., newsletters and emails) at both centers, and word-of-mouth was used to promote the study. A total of 146 HCPs from RVH and 94 from Waypoint submitted complete surveys, yielding a response rate of 6.6% for RVH and 14.0% for Waypoint.

## Results

### Demographic Characteristics

The majority of the participants were Caucasian, nurses, between 31 and 50 years of age, had <5 years of experience at their current job, lived with a partner, did not live with children, and worked on-site. There were no significant differences in these demographic categories between sites except for age (χ^2^(3, *N* = 240) = 10.7, *p* = 0.013) and profession (χ^2^(3, *N* = 239) = 25.3, *p* <0.001); however, the highest frequencies of these variables were the same for both sites: age 31–50 years and nursing profession. Please see Table 1.

**Table 1:** Demographics by site.

**Variable**	**Category**	**Waypoint**	**RVH**	**χ^2^**	***p*-value**
**Age**	<30	19 (20.21%)	44 (30.13%)	χ^2^(3, *N* = 240) = 10.7	0.013
	31–50	49 (52.12%)	80 (54.79%)		
	51–65	26 (27.65%)	19 (13.01%)		
	>65	0	3 (2.05%)		
**Profession**	Nursing	44 (46.80%)	97 (66.89%)	χ^2^(3, *N* = 239) = 25.3	0.000
	Physician	0	12 (8.275%)		
	Allied Health Professional	33 (35.10%)	27 (18.62%)		
	Other	17 (18.08%)	9 (6.20%)		
**Total experience**	<5 years	24 (25.53%)	42 (28.96%)	χ^2^(4, *N* = 239) = 1.3	0.86
	6–10	20 (21.27%)	33 (22.75%)		
	11–15	13 (13.82%)	26 (17.93%)		
	16–20	9 (9.57%)	18 (12.41%)		
	>20	28 (29.78%)	26 (17.93%)		
**Waypoint/RVH experience**	<5 years	40 (42.55%)	68 (46.57%)	χ^2^(4, *N* = 240) = 0.72	0.95
	6–10	21 (22.34%)	32 (21.91%)		
	11–15	10 (10.63%)	13 (8.904%)		
	16–20	11 (11.70%)	18 (12.32%)		
	>20	12 (12.76%)	15 (10.27%)		
Race/ethnicity^*^	Caucasian	84 (89.36%)	129 (87.75%)	χ^2^(4, *N* = 232) = 3.5	0.48
***N***		**94**	**146**		

* *Small numbers in race/ethnicity not presented to protect identity*.

### Relationships Between Study Variables

Relationships between the study variables using pooled data from both sites, assessed with Pearson's correlation tests, are presented in Table 2. As shown, all scales were significantly correlated with one another.

**Table 2:** Correlation between psychometric scales' scores.

		**CAS**	**ULS**	**WRQoL**	**CD-RISC**	**DASS(D)**	**DASS(A)**	**DASS(S)**
**CAS**	Pearson Correlation	1	0.351^**^	−0.374^**^	−0.195^**^	0.564^**^	0.672^**^	0.578^**^
	Sig. (2-tailed)		0.000	0.000	0.003	0.000	0.000	0.000
	*N*	239	233	234	234	230	230	230
**ULS**	Pearson Correlation	0.351^**^	1	−0.390^**^	−0.296^**^	0.580^**^	0.382^**^	0.456^**^
	Sig. (2-tailed)	0.000		0.000	0.000	0.000	0.000	0.000
	*N*	233	233	230	230	225	225	225
**WRQoL**	Pearson Correlation	−0.374^**^	−0.390^**^	1	0.480^**^	−0.538^**^	−0.463^**^	−0.541^**^
	Sig. (2-tailed)	0.000	0.000		0.000	0.000	0.000	0.000
	*N*	234	230	234	231	227	227	227
**CD-RISC**	Pearson Correlation	−0.195^**^	−0.296^**^	0.480^**^	1	−0.379^**^	−0.220^**^	−0.286^**^
	Sig. (2-tailed)	0.003	0.000	0.000		0.000	0.001	0.000
	*N*	234	230	231	235	226	226	226
**DASS(D)**	Pearson Correlation	0.564^**^	0.580^**^	−0.538^**^	−0.379^**^	1	0.686^**^	0.801^**^
	Sig. (2-tailed)	0.000	0.000	0.000	0.000		0.000	0.000
	*N*	230	225	227	226	230	230	230
**DASS(A)**	Pearson Correlation	0.672^**^	0.382^**^	−0.463^**^	−0.220^**^	0.686^**^	1	0.769^**^
	Sig. (2-tailed)	0.000	0.000	0.000	0.001	0.000		0.000
	*N*	230	225	227	226	230	230	230
**DASS(S)**	Pearson Correlation	0.578^**^	0.456^**^	−0.541^**^	−0.286^**^	0.801^**^	0.769^**^	1
	Sig. (2-tailed)	0.000	0.000	0.000	0.000	0.000	0.000	
	*N*	230	225	227	226	230	230	230

** *Correlation is significant at the 0.01 level (2-tailed)*.

### CAS and DASS-21 Score Distributions

**Figure 1:**
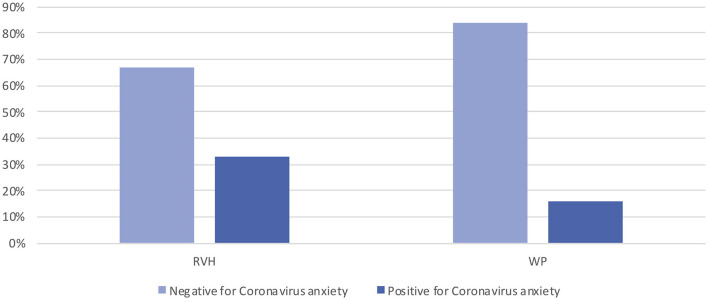
CAS scores by site.

When we examined the distributions of the CAS and DASS-21 data for the participating HCPs, we found that nearly half of the participants were asymptomatic on the DASS-21 sub-scales, but the remaining participants had symptoms ranging from mild to extremely severe. Similarly, more than one quarter (26.4%) of the respondents reported CAS scores in the clinical range. Please see Figure 1.

### Comparison of Psychometric Scales Between Departments at the General Hospital

Psychometric scale scores between the two sites are presented in Table 3. When we compared all the psychometric scores across medical and surgical departments at RVH using ANOVA, we found no significant difference in scale scores, with *p*-values ranging from 0.16 to 0.75.

**Table 3:** Psychometric scale scores.

	**Site**	
	**Waypoint**	**RVH**
**CAS**	*M* = 2.18	*M* = 3.70
	SD = 3.11	SD = 4.43
**ULS**	*M* = 16.53	*M* = 19.46
	SD = 13.93	SD = 15.84
**WRQoL**	*M* = 74	*M* = 76.99
	SD = 18.86	SD = 15.84
**CD-RISC**	*M* = 69.46	*M* = 69.73
	SD = 14.39	SD = 9.03
**DASS(D)**	*M* = 10.29	*M* = 11.42
	SD = 9.99	SD = 9.03
**DASS(A)**	*M* = 6.83	*M* = 8.21
	SD = 7.92	SD = 7.41
**DASS(S)**	*M* = 14.13	*M* = 15.16
	SD = 11.31	SD = 9.48

### Predictors of Resiliency and Quality of Life

We tested whether specific demographic variables could predict QoL and CD-RISC scores using multiple linear regression for each scale. Specifically, we tested whether type of HCP and living situation could predict these outcome variables. The overall regression model that tested predictors for WRQoL was significant (*R*^2^ = 0.03, *F*_(1, 232)_ = 6.7, *p* = 0.010). Being a nurse significantly predicted WRQoL scores (β = 5.7, *p* = 0.010). The regression model for CD-RISC was also significant (*R*^2^ = 0.02, *F*_(1, 233)_ = 4.7, *p* = 0.031). Being a nurse also predicted greater resilience scores (β = 4.0, *p* = 0.031).

## Discussion

This study assessed HCPs who worked during the COVID-19 pandemic at Waypoint, a large psychiatric hospital, and RVH, a large community acute care hospital, comparing the results on questionnaires of psychosocial functioning and stress resilence between sites. Our findings can be useful to leaders and policy writers to support the health of HCPs now and in the future, during future pandemic scenarios. Several findings emerged. First, HCPs displayed comparable levels of psychological symptoms at both sites. Second, there was no difference in scores among the different medical and surgical departments at RVH. Third, we found that being a nurse was a predictor for greater quality of life and stress resilience. We discuss each of these findings in turn.

Since data collection occurred at different time points at the two sites, it is notable that HCPs showed similar scores on the psychometric assessments by site. At the time of data collection at Waypoint, there were no positive COVID-19 cases in either staff or patients. Past reports suggest that the amount of time spent with infectious patients may create differences in the way epidemic outbreaks affect the psychological wellbeing of HCPs (32). Based on this information, we would have expected that psychological distress was significantly greater in the HCPs working at RVH, given that these HCPs had more exposure to COVID-19. To facilitate a more robust comparison between sites, larger sample sizes and sampling at the same wave of the pandemic would have been ideal.

The absence of large differences in relatively high scale scores between sites and between clinical specialties points to a pervasive effect on the psychosocial well-being of HCPs, who are an already at-risk population (10–12). Promoting effective inter-professional relationships and strong communication have been identified as important factors in resilience building strategies (33). HCPs need to be provided with effective supportive interventions, regardless of their location or specialty.

Fear of contagion and fear of infecting family, friends, and colleagues, as well as uncertainty about the virus and stigmatization of infected individuals, have been described as factors that can affect the mental health of HCPs (34). HCPs working in acute and critical care settings have been described as a population vulnerable to burnout during epidemic outbreaks, with anxiety disorders and care of patients with COVID-19 listed as factors that may influence the occurrence of burnout (35). Anxiety-related symptoms are common in HCPs working with COVID-19 patients according to the literature reviewed in this paper and should be one of the main focus points in interventions aimed at improving the mental health of HCPs.

The DASS-21 subscale scores were similar between sites. When we grouped the data using the cut scores suggested by the scale's authors, we found that nearly half of the sample was asymptomatic at both sites for all the subscales but that the other half presented scores ranging from mild to extremely severe in all the subscales. A similar phenomenon was observed for the CAS scores, where more than one-quarter of the respondents scored positive for coronavirus anxiety. These results imply that at the time our data were collected, participants were generally resilient and coping well, but there is a group of HCPs at both sites who were struggling. This finding suggests that to optimize the effectiveness of resilience-building programs, developers and policymakers ought to carefully assess their workforce, identifying individuals at elevated risk who may need special attention (36). Research conducted during the COVID-19 pandemic has shown that HCPs are experiencing burnout (15, 16); higher levels of depression, anxiety, and stress (35, 37); insomnia; and somatization and obsessive-compulsive symptoms (19, 38), which concurs with our own findings.

Finally, regression models indicated that nurses scored higher in quality of life and greater resilience compared with other HCPs. Nurses may have received relatively more training on interprofessional collaboration (39, 40), empathy training (41), and leadership experience (42–44) than their peers, which may have buffered them against the effects of the pandemic. There is evidence to suggest that when nurses develop their own personal resilience, they can reduce their vulnerability to workplace adversity and thus improve the overall healthcare setting (45). Resilience-building should be incorporated into nursing and other HCPs' education. The professional characteristics of nursing training make nurses natural leaders to implement strategies aimed at the protection of HCPs in crisis situations.

The findings from this study can inform policymakers and senior management at hospitals to carefully consider the psychosocial functioning of their HCPs during crisis situations. While not all HCPs have been negatively affected during the COVID-19 pandemic, some individuals are struggling, and these HCPs need to be quickly identified and provided with targeted interventions. In general, allied HCPs and physicians fared worse than nursing staff, which suggests that they should be scrutinized more carefully. It is also clear that as the pandemic worsened, HCPs understandably became more anxious about COVID-19, which makes delivering interventions during periods of heightened transmission all the more important.

There is evidence from other trauma-exposed populations, such as firefighters or military personnel, that supervisor training is beneficial in reducing work-related sickness absence (46). Moreover, peer support interventions improve the likelihood of at-risk individuals seeking help from mental health services (47). Implementing programs and assessments aimed at identifying at-risk HCPs is important when formulating resilience-building programs (36), and early implementation is crucial, as there is evidence of deteriorating depression and anxiety among HCPs as the COVID-19 pandemic evolved (48). Our study adds to this information by showing that expression of psychological symptoms was similar in two different hospital settings at different phases of the pandemic.

There is also evidence that patient experience shapes HCPs' experience, and improving patient circumstances can have a significant effect on the mental health and general well-being of HCPs (49). Acknowledging the experiences of at-risk individuals is of paramount importance, as their circumstances must be adressed in strategic initiatives targeted at bolstering strong psychosocial functioning before mental health deterioration becomes a long lasting problem in the workforce (50, 51).

Previous research has shown that the SARS outbreak in Toronto in 2003 negatively affected HCPs' mental health (34). Similarly, during the H1N1 influenza outbreak in 2009, HCPs reported high levels of anxiety and exhaustion (32, 52, 53). Now the third pandemic has occurred in <20 years. A growing understanding of the lasting stresses in HCPs and the need for interventions aimed at strengthening the resilience and ability to cope in HCPs is mounting. Resilience is a dynamic, evolving process of positive attitudes and effective strategies (33), and it can be challenging to promote this process during a time of crisis. Hence, it is important to implement policies aimed at increasing resilience and coping mechanisms before the next crisis arrives; during the crisis, a step-wise and personalized approach should be considered (54).

Several study limitations must be noted. First, our study may have been prone to sampling bias in that respondents self-selected to participate. Our response rates at RVH and Waypoint were 6.6 and 14.0%, respectively, lower than a reported average of 46% for online surveys detailed in a recent systematic review (55). Thus, non-response bias may have impacted the generalizability of results. To help explain these results, it is possible that more symptomatic individuals chose not to participate in the surveys, because their symptoms discouraged participation. Halbesleben and Whitman (56) suggest that benchmarking findings against other published data and examining whether descriptive statistics for comparable measures are consistent with previously published studies is a helpful technique. When we compared our sample size to those reported in other published studies employing the same validated scales, we noted comparable sample sizes. Moreover, online surveys remain the preferred method as a cost-effective means of collecting information on healthcare delivery (57, 58). Another limitation is the relatively small sample size. We acknowledge that it is possible that our study was underpowered to detect differences between sites. Larger samples would have allowed us to conduct statistical analyses to compare sites. However, since the sample size was relatively low and because we were sampling sites at different times during the pandemic, we elected not to conduct statistical analyses but simply report descriptive data.

Although our sample was disproportionately white and female with higher nursing representation, these variables reflect the demographics of the institutions we sampled. However, results may not be generalizable to other centers where nurses are fewer and there are more male employees. Another limitation is that we did not have baseline data on possible mental health disorders in the respondents who completed the survey. This information could have affected the results obtained. As noted, we also sampled our participants at Waypoint and RVH at different time points during the pandemic. Future studies that compare the psychosocial functioning of HCPs at multiple institutions may choose to sample their participants contemporaneously to avoid this bias.

The results of this study provide the basis for several recommendations. First, a crisis situation like a pandemic can have widespread effects on all aspects of clinical practice, including the mental health of HCPs (59). Managers should actively monitor the well-being of HCPs and create policies and interventions aimed at building resilience in HCPs before a new crisis emerges. Second, given the wide distribution of symptom severity observed in the DASS-21, it is also important to identify individuals at-risk during the crisis to provide targeted interventions: evidence-based staff support should be made available to all HCPs during a widespread crisis like the COVID-19 pandemic. Third, nursing professionals appear more resilient, suggesting that they have learned skills and have had experiences that make them good candidates to lead and deliver interventions for the general HCP population. Finally, COVID-19 has acted as a catalyst for changes in the way that HCPs conduct clinical duties and carry out administrative tasks. In conclusion, it would behoove researchers to explore ways, in the broader context of institutional clinical practice, that may influence the formulation of management strategies to preserve the well-being of all HCPs during times of crisis and accelerated change.

## Data Availability Statement

The raw data supporting the conclusions of this article will be made available by the authors, without undue reservation.

## Ethics Statement

The studies involving human participants were reviewed and approved by the Research Ethics Boards of the Royal Victoria Regional Health Centre, Waypoint Centre for Mental Health Care, and Georgian College. The participants provided their written consent to participate in this study.

## Author Contributions

DA, JL, JM, and NJK were responsible for the study design, data collection, conceptualization, and analysis. DA and NJK were responsible for drafting the initial version of the manuscript. All authors provided critical revisions to the manuscript and approved the final version.

## Funding

This study received funding through the NSERC College and Community Innovation Program (CCI): Applied Research Rapid Response to COVID-19 (file COVPJ 554317–20), awarded to NJK and JL.

## Conflict of Interest

The authors declare that the research was conducted in the absence of any commercial or financial relationships that could be construed as a potential conflict of interest.

## Publisher's Note

All claims expressed in this article are solely those of the authors and do not necessarily represent those of their affiliated organizations, or those of the publisher, the editors and the reviewers. Any product that may be evaluated in this article, or claim that may be made by its manufacturer, is not guaranteed or endorsed by the publisher.
